# Alcohol consumption, cigarette smoking and cancer of unknown primary risk: Results from the Netherlands Cohort Study

**DOI:** 10.1002/ijc.33328

**Published:** 2020-10-20

**Authors:** Karlijn E. P. E. Hermans, Piet A. van den Brandt, Caroline Loef, Rob L. H. Jansen, Leo J. Schouten

**Affiliations:** ^1^ Department of Epidemiology GROW‐School for Oncology and Developmental Biology, Maastricht University Maastricht The Netherlands; ^2^ Department of Research Comprehensive Cancer Organisation the Netherlands Utrecht The Netherlands; ^3^ Department of Internal Medicine, Medical Oncology GROW‐School for Oncology and Developmental Biology, Maastricht University Medical Center+ Maastricht The Netherlands

**Keywords:** alcohol, cancer of unknown primary (CUP), prospective cohort study, smoking

## Abstract

Cancer of unknown primary (CUP) is a metastasised malignancy with no identifiable primary tumour origin. Despite the frequent occurrence and bleak prognosis of CUP, research into its aetiology is scarce. Our study investigates alcohol consumption, tobacco smoking and CUP risk. We used data from the Netherlands Cohort Study, a cohort that includes 120 852 participants aged 55 to 69 years, who completed a self‐administered questionnaire on cancer risk factors at baseline. Cancer follow‐up was established through record linkage to the Netherlands Cancer Registry and Dutch Pathology Registry. After 20.3 years of follow‐up, 963 CUP cases and 4288 subcohort members were available for case‐cohort analyses. Multivariable‐adjusted hazard ratios (HRs) were calculated using proportional hazard models. In general, CUP risk increased with higher levels of alcohol intake (*P*_trend_ = .02). The association was more pronounced in participants who drank ≥30 g of ethanol per day (HR: 1.57, 95% confidence interval [CI]: 1.20‐2.05) compared to abstainers. Current smokers were at an increased CUP risk (HR: 1.59, 95% CI: 1.29‐1.97) compared to never smokers. We observed that the more the cigarettes or the longer a participant smoked, the higher the CUP risk was (*P*_trend_ = .003 and *P*_trend_ = .02, respectively). Interaction on additive scale was found for participants with the highest exposure categories of alcohol consumption and cigarette smoking frequency and CUP risk. Our findings demonstrate that alcohol consumption and cigarette smoking are associated with increased CUP risk. Lifestyle recommendations for cancer prevention regarding not drinking alcohol and avoiding exposure to smoking are therefore also valid for CUP.

## Abstract 2

**What's new?**

Little is known about factors that raise the risk of cancer of unknown primary (CUP), in which metastases have no identifiable primary tumor origin. This prospective study examined possible associations between CUP and alcohol consumption and cigarette smoking, using sex‐stratified analyses and assessing variables such as smoking frequency and duration. Analyses show that higher alcohol consumption, current cigarette smoking, more cigarettes smoked, and longer smoking duration are associated with increased CUP risk. The data highlight the significance of lifestyle factors in CUP risk and suggest that lifestyle recommendations regarding alcohol intake and smoking are relevant to CUP prevention.

**Abbreviations**
CI: confidence interval
CUP: cancer of unknown primary
EPIC: European Prospective Investigation into Cancer and Nutrition
HR: hazard ratio
NCR: Netherlands Cancer Registry
NLCS: Netherlands Cohort Study
OR: odds ratio
PALGA: Dutch Pathology Registry

## 1 INTRODUCTION

Cancer of unknown primary (CUP) is a heterogeneous group of metastasised malignancies with no identifiable primary tumour origin.^1^, ^2^ Cancer treatment, if any, is generally based on the primary tumour origin, which makes treating CUP challenging. Another complexity is the absence of consensus on a CUP definition. Due to the use of different definitions globally, it is difficult to compare this entity.^3^ In the Netherlands, the cancer clinical practice guidelines advise to use the definition “CUP” if the patient has a metastasis of an unknown primary tumour origin, based on a cytological and/or histological proven metastasis of a cancer.^4^ In 2018, CUP accounted for approximately 1300 incident cases in the country, this contributed to almost 2% of all cancers as registered by the Netherlands Cancer Registry (NCR).^5^, ^6^

CUP occurrence is equal in both sexes. On average, patients are aged 74 years at diagnosis.^5^ The disease primarily concerns adenocarcinoma (ca. 60%) and undifferentiated carcinoma (ca. 20%), with the most common metastatic sites of presentation being the liver (ca. 40%) and lymph nodes (ca. 20%).^2^, ^7^ In the Netherlands, the overall median survival for patients with a CUP diagnosis between 2010 and 2012 was 1.7 months.^2^ Despite the frequent occurrence and bleak prognosis of CUP, research into its aetiology is particularly scarce. Potential CUP risk factors that have previously been identified include diabetes, family history of cancer, waist circumference and smoking.^8^, ^9^, ^10^, ^11^

Lifestyle recommendations for cancer prevention have described both alcohol consumption and tobacco smoking as modifiable cancer‐risk factors. These recommendations advise against drinking alcohol and avoiding exposure to smoking.^12^, ^13^, ^14^, ^15^ To date, few studies have investigated the association between alcohol consumption, tobacco smoking and CUP risk.^10^, ^11^, ^16^ Two prospective cohort studies investigated alcohol consumption and did not observe an association with CUP risk.^10^, ^11^ Three studies demonstrated a strong association between smoking and CUP risk.^10^, ^11^, ^16^ None of these studies, however, assessed the association between smoking duration and CUP risk, and one did not investigate smoking frequency.^16^ Therefore, we aimed to investigate the association between alcohol consumption, tobacco smoking and the development of CUP in greater depth. We hypothesise that (a) CUP risk is higher in participants with a high intake of alcoholic drinks, (b) CUP risk is higher in participants who smoke and (c) there is a multiplicative or additive interaction effect between alcohol consumption, tobacco smoking and CUP risk.

## 2 MATERIALS AND METHODS

### 2.1 Design and study population

The Netherlands Cohort Study (NLCS) was started on 17 September 1986 and includes 120 852 participants aged 55 to 69 years at baseline from 204 Dutch municipalities. Data processing and analyses were based on the nested case‐cohort design. Cases were derived from the full cohort while the number of person‐years at risk for the full cohort was estimated from a subcohort of 5000 participants who were randomly sampled from the full cohort at baseline.^17^

### 2.2 Outcome measure

In our study, CUP is defined as a metastasised epithelial malignancy with no identifiable primary tumour origin after cytological and/or histological verification during a patient's lifetime. This CUP definition only includes epithelial malignancies (ICD‐O‐3: M‐8000—M‐8570), which excludes, for example, sarcoma, lymphoma, mesothelioma and melanoma.

### 2.3 Follow‐up

Cancer follow‐up was established through annual record linkage of the full cohort with the NCR and the Dutch Pathology Registry (PALGA) to identify CUP cases within the NLCS.^18^ Information regarding the site of metastasis was obtained from the NCR, but data were only partially available and, therefore, information was requested and retrieved from PALGA pathology excerpts. These pathology excerpts were also used to determine whether cytological and/or histological confirmed cases had been correctly categorised in the data received from the NCR.

**FIGURE 1:**
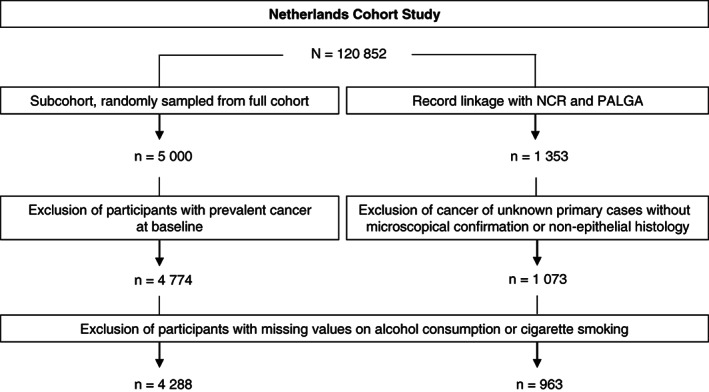
Flow diagram of subcohort members and Cancer of Unknown Primary cases in the Netherlands Cohort Study on whom analyses are based

After 20.3 years of follow‐up (17 September 1986 until 31 December 2006), data were available for a total of 1353 potential CUP cases. After excluding those cases without microscopical confirmation or nonepithelial histology, a total of 1073 CUP cases remained. These CUP cases were further subdivided: according to histology (adenocarcinoma, undifferentiated carcinoma, squamous cell carcinoma, neuroendocrine carcinoma and other carcinoma); according to number of metastases (multiple metastases of the same type were counted as one metastatic site, for example, bone metastases in hip and vertebra were counted as one); according to localisation of metastasis (up to four localisations); and according to survival duration (≤1 and >1 year after diagnosis). The subcohort consisted of 4774 participants after excluding members who reported a history of cancer (except for skin cancer) at baseline. Participants were also excluded when there were missing values on alcohol consumption or cigarette smoking. As a result, 963 CUP cases and 4288 subcohort members were available for investigation (see Figure 1).

### 2.4 Questionnaire data

All cohort members completed a self‐administered questionnaire, which included detailed questions on alcohol consumption, tobacco smoking and other cancer risk factors. Alcohol consumption was measured over the year preceding baseline and was addressed by questions on beer, red wine, white wine, sherry, other fortified wines, liqueurs and liquor. Frequency of alcohol consumption ranged from “never” to “6 to 7 times per week” and information on the number of glasses consumed per day. Participants who reported “never” or consumed “less than once per month” were considered abstainers. Four items from the questionnaire (red wine, white wine, sherry and liqueurs) were combined into one single wine variable since these items were substantially correlated, and separate analysis would have resulted in small numbers of subjects within each stratum. Mean daily alcohol consumption was calculated by using the computerised Dutch food composition table.^19^ Based on pilot study data, standard glass sizes were defined as 200 mL for beer, 105 mL for wine, 80 mL for sherry and 45 mL for both liqueurs and liquor, corresponding to 8, 10, 11, 7 and 13 g of ethanol, respectively. The food frequency questionnaire was validated against a 9‐day diet record. The Spearman correlation coefficient between alcohol consumption as assessed by the questionnaire and that estimated by the diet record was 0.89 for all subjects and 0.85 for alcohol consumers.^20^, ^21^ Tobacco smoking was addressed through questions on baseline smoking status and the ages at first exposure and last (if stopped) exposure to smoking. Questions were also asked about smoking frequency and smoking duration (excluding stopping periods) for cigarette, cigar and pipe smokers. As the vast majority of smoking subcohort members were cigarette smokers, analyses were restricted to that particular group. Based on the questionnaire data, the following cigarette smoking variables were constructed: cigarette smoking status (never, ex, current); frequency (cigarettes per day); duration (years); and time since smoking cessation (years). Participants who indicated that they had never smoked cigarettes were considered never smokers. Time since smoking cessation was calculated as age at baseline minus age at smoking cessation. To avoid collinearity problems, smoking frequency and smoking duration were centred as proposed by Leffondré et al.^22^

### 2.5 Statistical analysis

Person‐years at risk were calculated from baseline (17 September 1986) until CUP diagnosis, death, emigration, loss to follow‐up or end of follow‐up (31 December 2006), whichever occurred first. Patient characteristics were presented for CUP cases and stratified for histological and cytological confirmation. General characteristics were presented for subcohort members and CUP cases with frequencies (percentages), for categorical variables and means, including SDs for continuous variables. Alcohol consumption was measured as a continuous variable with 10 g of ethanol per day increments and in categories: abstainers, >0 to <5, 5 to <15, 15 to <30 and ≥30 g of ethanol per day. Cigarette smoking was assessed based on status, frequency, duration and time since smoking cessation. Cigarette smoking status was classified as never, ex or current. Cigarette smoking frequency was measured as a continuous variable with 10 cigarettes smoked per day increments and in categories: never smokers, >0 to <10, 10 to <20 and ≥20 cigarettes smoked per day. Cigarette smoking duration was investigated as a continuous variable with cigarette smoking increments of 10 years and in categories never smokers, >0 to <20, 20 to <40 and ≥40 years smoked. Time since smoking cessation was categorised as never smokers, stopped smoking ≥20 years, 10 to <20 years, >0 to <10 years and current smokers.

Alcohol consumption and cigarette smoking were mutually adjusted in the analyses. Predefined confounders were age at baseline (years; continuous) and sex (male/female). Potential confounders were body mass index (BMI) at baseline (kg/m^2^), nonoccupational physical activity (≤30 min/d, >30 to 60 min/d, >60 to 90 min/d and >90 min/d), socioeconomic status (highest level of education) and history of cancer in a first degree relative (yes/no). Variables were considered a confounder if they are not an independent risk factor, not associated with the investigated exposure variables, and if the HR did not change by >10% when adding the potential confounder to the model. Accordingly, none of the potential confounders were included in the final model.

Cox proportional hazards models were used to evaluate associations of alcohol consumption, cigarette smoking and CUP risk. Associations were estimated using age‐ and sex‐adjusted, and multivariable adjusted hazard ratios (HRs) with 95% confidence intervals (CIs). Attributable risks were calculated for alcohol consumption and cigarette smoking. SEs were calculated using the robust Huber‐White sandwich estimator to account for additional variance introduced by sampling from the full cohort.^23^ The proportional hazards assumption was tested using the scaled Schoenfeld residuals,^24^ and by visual inspection of log‐minus‐log (LML) survival curves. If there was an indication that the assumption had been violated, a time‐varying covariate for that variable was added to investigate in the model. Tests for dose‐response trends were assessed by fitting ordinal exposure variables as continuous terms. Wald tests and cross‐product terms were used to evaluate potential multiplicative interaction between alcohol consumption, sex and CUP; between cigarette smoking, sex and CUP; and between alcohol consumption, cigarette smoking frequency and CUP. Interaction on additive scale between alcohol consumption, cigarette smoking frequency and CUP was calculated using the relative excess risk due to interaction (RERI). Analyses were conducted using Stata version 15. *P* values were considered statistically significant if *P* < .05.

Sensitivity analyses were conducted with restriction to histologically verified CUP cases, and after excluding the first 2 years and the first 5 years of follow‐up to check for potential reverse causality bias.

## 3 RESULTS

Data analysis was based on 963 CUP cases and 4288 subcohort members for whom the data on alcohol consumption and cigarette smoking were complete. Overall, CUP patients were on average aged 73 years at diagnosis, the majority of whom were men (62.6%) and most cases were histologically verified (71.3%) (see Table 1). The most common histological subtype was adenocarcinoma (64.8%). The majority of patients were registered with a single organ metastasis (80.3%), and the most frequent metastatic site of presentation was the liver (37.9%). Most patients had died within a year after CUP diagnosis (73.4%).

**TABLE 1:** Patient characteristics of CUP cases in the Netherlands Cohort Study

	CUP cases					
	Overall (n = 963)		Histologically confirmed (n = 687)		Cytologically confirmed (n = 276)	
	n	(%)	n	(%)	n	(%)
Age at baseline (y), %						
55 to 59	288	29.9	224	32.6	64	23.2
60 to 64	372	38.6	259	37.7	113	40.9
65 to 69	303	31.5	231	29.7	99	35.9
Age at diagnosis (y), mean (SD)						
Overall	73.3 (6.4)		72.8 (6.4)		74.7 (6.1)	
Sex, %						
Men	603	62.6	440	64.1	163	59.1
Women	360	37.4	247	36.0	113	40.9
Histology, %						
Adenocarcinoma	624	64.8	440	64.1	184	66.7
Undifferentiated carcinoma	192	19.9	133	19.4	59	21.4
Squamous cell carcinoma	47	4.9	38	5.5	9	3.3
Neuroendocrine carcinoma	35	3.6	32	4.7	3	1.1
Other carcinoma	65	6.8	44	6.4	21	7.6
Number of metastatic sites, %						
1	773	80.3	534	77.7	239	86.6
2+	166	17.2	140	20.4	26	9.4
Most frequent metastatic site of presentation, %						
Liver	365	37.9	307	44.7	58	21.0
Lymph node	158	16.4	118	17.2	40	14.5
Peritoneum	160	16.6	99	14.4	61	22.1
Bone	149	15.5	125	18.2	24	8.7
Lung	78	8.1	43	6.3	35	12.7
Survival status, %						
Survival ≤1 y after diagnosis	707	73.4	496	72.2	211	76.5
Survival >1 y after diagnosis	256	26.6	191	27.8	65	23.6

Abbreviation: CUP, cancer of unknown primary.

Overall, CUP cases were more often alcohol consumers with a substantially higher ethanol intake (≥30 g of ethanol) in comparison to subcohort members (16.6% vs 9.0%) (see Table 2). This higher intake was especially evident in male CUP patients of whom 23.9% drank ≥30 g of ethanol per day on average in comparison to 14.7% of men in the subcohort. With respect to cigarette smoking, CUP cases were generally more often current smokers (37.8%) and less often never smokers (27.5%) in comparison to subcohort members (27.6% and 36.9%, respectively). Again, male CUP patients, in particular, were more often current smokers 44.9% in comparison to 34.8% of the men in the subcohort. In addition, the number of cigarettes smoked per day and smoking duration in years was higher for CUP patients on average in comparison to those of the subcohort members.

**TABLE 2:** Characteristics of CUP cases and subcohort members in the Netherlands Cohort Study

	Subcohort members			CUP cases		
	Total (M + F) (n = 4288)	Men only (n = 2110)	Women only (n = 2178)	Total (M + F) (n = 963)	Men only (n = 603)	Women only (n = 360)
Exposure variables and potential confounders	n (%)	n (%)	n (%)	n (%)	n (%)	n (%)
Age at baseline (y)						
55 to 59	1664 (38.8)	818 (38.8)	846 (38.8)	288 (29.9)	187 (31.0)	101 (28.1)
60 to 64	1461 (34.1)	730 (34.6)	731 (33.6)	372 (38.6)	231 (38.3)	141 (39.2)
65 to 69	1163 (27.1)	562 (26.6)	601 (27.6)	303 (31.5)	185 (30.7)	118 (32.8)
Sex	4288 (100)	2110 (49.2)	2178 (50.8)	963 (100)	603 (62.6)	360 (37.4)
Alcohol consumption						
Ethanol intake (g/d)						
Abstainers	1024 (23.9)	313 (14.8)	711 (32.6)	186 (19.3)	71 (11.8)	115 (31.9)
<5	1228 (28.6)	439 (20.8)	789 (36.2)	247 (25.7)	115 (19.1)	132 (36.7)
5 to <15	979 (22.8)	576 (27.3)	403 (18.5)	217 (22.5)	146 (24.2)	71 (19.7)
15 to <30	672 (15.7)	471 (22.3)	201 (9.2)	153 (15.9)	127 (21.1)	26 (7.2)
≥30	385 (9.0)	311 (14.7)	74 (3.4)	160 (16.6)	144 (23.9)	16 (4.4)
Ethanol intake (10 g of ethanol per day increments), mean (SD)^a^	2.5 (1.5)	2.7 (1.7)	2.1 (1.1)	3.0 (1.9)	3.2 (1.9)	2.2 (1.2)
Cigarette smoking						
Cigarette smoking status						
Never smokers	1584 (36.9)	291 (13.8)	1293 (59.4)	265 (27.5)	61 (10.1)	204 (56.7)
Ex‐smokers	1521 (35.5)	1085 (51.4)	436 (20.0)	334 (34.7)	271 (44.9)	63 (17.5)
Current smokers	1183 (27.6)	734 (34.8)	449 (20.6)	364 (37.8)	271 (44.9)	93 (25.8)
Frequency of cigarette smoking (N/d), mean (SD)^b^	15.7 (10.1)	17.3 (10.5)	12.3 (8.1)	17.8 (10.4)	19.0 (10.7)	13.4 (7.8)
Duration of cigarette smoking (y), mean (SD)^b^	31.9 (12.1)	33.8 (11.6)	28.0 (12.2)	35.6 (11.6)	37.0 (10.8)	30.7 (12.7)
Cigarette smoking cessation (y), mean (SD)^b^	14.5 (9.7)	14.8 (9.4)	13.8 (10.5)	12.9 (9.2)	12.7 (9.0)	13.3 (10.2)
Other risk factors						
Body mass index at baseline (kg/m^2^), mean (SD)	25.0 (3.1)	25.0 (2.6)	25.1 (3.5)	25.0 (3.0)	24.9 (2.7)	25.1 (3.5)
Nonoccupational physical activity (min/d)						
≤30	908 (21.5)	390 (18.7)	518 (24.2)	204 (21.5)	108 (18.1)	96 (27.1)
>30 to 60	1318 (31.2)	633 (30.4)	685 (31.9)	291 (30.6)	181 (30.4)	110 (31.1)
>60 to 90	879 (20.8)	396 (19.0)	483 (22.5)	170 (17.9)	93 (15.6)	77 (21.8)
>90	1122 (26.5)	663 (31.8)	459 (21.4)	285 (30.0)	214 (35.9)	71 (20.1)
Level of education (years of education)						
Primary	1257 (29.5)	535 (25.5)	722 (33.3)	271 (28.5)	139 (23.2)	132 (37.3)
Lower vocational	937 (22.0)	429 (20.4)	508 (23.4)	204 (21.4)	117 (19.6)	87 (24.6)
Secondary and medium vocational	1483 (34.8)	739 (35.2)	744 (34.3)	341 (35.8)	234 (39.1)	107 (30.2)
University and higher vocational	590 (13.8)	396 (18.9)	194 (9.0)	136 (14.3)	108 (18.1)	28 (7.9)
History of cancer in a first degree relative						
Yes	1694 (45.2)	821 (43.3)	873 (47.2)	410 (48.5)	264 (48.3)	146 (48.8)

Abbreviation: CUP, cancer of unknown primary.

^a^ In consumers only.

^b^ In users only.

In general, a higher ethanol intake was associated with an increased CUP risk (*P*_trend_ = .02; see Table 3). Participants who reported consuming ≥30 g of ethanol per day were compared to abstinence, at the highest risk of developing CUP (multivariable adjusted HR: 1.57, 95% CI: 1.20‐2.05). The attributable risk for alcohol consumption on CUP risk was 4% (95% CI: 2%‐6%). No multiplicative interaction was observed between alcohol consumption categories, sex, and CUP risk (*P*_interaction_ = .86).

**TABLE 3:** Hazard ratios and 95% CIs for alcohol consumption and CUP risk in the Netherlands Cohort Study

	Subcohort members		CUP cases				
	Categorical median	Person time at risk (y)	Cases	Age and sex‐adjusted^a^		Multivariable adjusted^b^	
			n	HR	95% CI	HR	95% CI
Ethanol intake (g/d)							
Overall							
Abstainers	0	17 180	186	1	Reference	1	Reference
>0 to <5	2	21 482	247	1.06	(0.86‐1.31)	1.10	(0.88‐1.36)
5 to <15	9	16 214	217	1.11	(0.88‐1.38)	1.13	(0.90‐1.41)
15 to <30	22	11 159	153	1.05	(0.82‐1.34)	0.97	(0.76‐1.25)
≥30	40	6307	160	1.82	(1.41‐2.36)	1.57	(1.20‐2.05)
*p* for trend^c^				<.001		.02	
Continuous, 10 g of ethanol per day increments		72 342	963	1.12	(1.07‐1.17)	1.08	(1.03‐1.13)
Men							
Abstainers	0	4755	71	1	Reference	1	Reference
>0 to <5	2	7051	115	1.16	(0.82‐1.62)	1.19	(0.84‐1.67)
5 to <15	9	9057	146	1.17	(0.85‐1.62)	1.20	(0.86‐1.67)
15 to <30	22	7637	127	1.18	(0.85‐1.64)	1.08	(0.77‐1.51)
≥30	41	5029	144	2.00	(1.43‐2.79)	1.71	(1.21‐2.41)
*p* for trend^c^				<.001		.01	
Continuous, 10 g of ethanol per day increments		33 528	603	1.13	(1.08‐1.18)	1.09	(1.03‐1.14)
Women							
Abstainers	0	12 425	115	1	Reference	1	Reference
>0 to <5	2	14 431	132	1.00	(0.76‐1.31)	1.03	(0.78‐1.35)
5 to <15	9	7157	71	1.09	(0.79‐1.51)	1.09	(0.79‐1.51)
15 to < 30	21	3522	26	0.83	(0.53‐1.31)	0.80	(0.50‐1.27)
≥30	37	1279	16	1.51	(0.84‐2.72)	1.28	(0.70‐2.35)
*p* for trend^c^				.63		.92	
Continuous, 10 g of ethanol per day increments		38 814	360	1.04	(0.91‐1.18)	1.00	(0.88‐1.14)

Abbreviations: CI, confidence interval; CUP, cancer of unknown primary; HR, hazards ratio.

^a^ Analyses were adjusted for age at baseline (years) and sex.

^b^ Multivariable analyses were additionally adjusted for current cigarette smoking, frequency (continuous; centred) and duration (continuous; centred).

^c^ Tests for dose‐response trends were assessed by fitting ordinal variables as continuous terms in the Cox proportional hazards model.

Current smokers were at an increased risk of developing CUP (multivariable‐adjusted HR: 1.59, 95% CI: 1.29‐1.97) compared to never smokers (see Table 4). For cigarette smoking status, the attributable risk for CUP risk was 6% (95% CI: 4%‐8%). After stratification for sex, we observed that CUP risk was the highest for current smokers compared to never smokers, in both men and women (HR: 1.64, 95% CI: 1.16‐2.31 and HR: 1.62, 95% CI: 1.21‐2.16, respectively).

**TABLE 4:** Hazard ratios and 95% CIs for cigarette smoking and CUP risk in the Netherlands Cohort Study

	Subcohort members		CUP cases				
	Categorical median	Person time at risk (y)	Cases	Age and sex‐adjusted^a^		Multivariable adjusted^b^	
			n	HR	95% CI	HR	95% CI
Cigarette smoking status^c^							
Overall							
Never smokers		28 472	265	1	Reference	1	Reference
Ex‐smokers		25 427	334	1.09	(0.90‐1.33)	1.19	(0.97‐1.47)
Current smokers		18 443	364	1.85	(1.53‐2.24)	1.59	(1.29‐1.97)
*p* for trend^d^				<.001		<.001	
Men							
Never smokers		5026	61	1	Reference	1	Reference
Ex‐smokers		17 558	271	1.30	(0.95‐1.78)	1.24	(0.90‐1.70)
Current smokers		10 945	271	2.27	(1.66‐3.11)	1.64	(1.16‐2.31)
*p* for trend^d^				<.001		.004	
Women							
Never smokers		23 446	204	1	Reference	1	Reference
Ex‐smokers		7869	63	0.99	(0.73‐1.34)	1.21	(0.87‐1.68)
Current smokers		7498	93	1.59	(1.21‐2.09)	1.62	(1.21‐2.16)
*p* for trend^d^				.003		.001	
Cigarette smoking frequency (N/d)^e^							
Overall							
Never smokers	0	28 472	265	1	Reference	1	Reference
>0 to <10	5	12 521	119	0.94	(0.74‐1.19)	0.86	(0.65‐1.14)
10 to <20	12	15 252	264	1.59	(1.28‐1.97)	1.27	(1.00‐1.62)
≥20	20	16 097	315	1.83	(1.48‐2.25)	1.42	(1.13‐1.80)
*p* for trend^d^				<.001		.003	
Continuous, 10 cigarettes per day increments		72 342	963	1.21	(1.12‐1.30)	1.17	(1.08‐1.27)
Men							
Never smokers	0	5026	61	1	Reference	1	Reference
>0 to <10	5	5488	68	1.02	(0.69‐1.50)	0.91	(0.60‐1.37)
10 to <20	12	10 622	205	1.64	(1.19‐2.27)	1.27	(0.90‐1.78)
≥20	20	12 392	269	1.98	(1.44‐2.70)	1.49	(1.07‐2.07)
*p* for trend^d^				<.001		.004	
Continuous, 10 cigarettes per day increments		33 528	603	1.21	(1.12‐1.31)	1.17	(1.07‐1.28)
Women							
Never smokers	0	23 446	204	1	Reference	1	Reference
>0 to <10	4	7033	51	0.88	(0.64‐1.23)	0.86	(0.56‐1.30)
10 to <20	12	4630	59	1.67	(1.21‐2.31)	1.45	(0.96‐2.19)
≥20	20	3704	46	1.60	(1.12‐2.28)	1.34	(0.88‐2.03)
*p* for trend^d^				.001		.23	
Continuous, 10 cigarettes per day increments		38 814	360	1.22	(1.00‐1.49)	1.19	(0.97‐1.45)
Cigarette smoking duration (y)^f^							
Overall							
Never smokers	0	28 472	265	1	Reference	1	Reference
>0 to <20	13	8435	82	0.96	(0.73‐1.28)	0.95	(0.71‐1.27)
20 to <40	30	21 338	279	1.24	(1.02‐1.51)	1.07	(0.86‐1.33)
≥40	43	14 097	337	2.03	(1.65‐2.49)	1.45	(1.09‐1.94)
*p* for trend^d^				<.001		.02	
Continuous, 10 y increments		72 342	963	1.35	(1.24‐1.47)	1.18	(1.07‐1.30)
Men							
Never smokers	0	5026	61	1	Reference	1	Reference
>0 to <20	14	4398	45	0.91	(0.59‐1.39)	0.86	(0.56‐1.33)
20 to <40	30	13 333	215	1.42	(1.03‐1.95)	1.20	(0.87‐1.66)
≥40	44	10 772	282	2.20	(1.61‐3.01)	1.49	(1.02‐2.19)
*p* for trend^d^				<.001		.01	
Continuous, 10 y increments		33 528	603	1.40	(1.26‐1.54)	1.23	(1.08‐1.39)
Women							
Never smokers	0	23 446	204	1	Reference	1	Reference
>0 to <20	11	4037	37	1.14	(0.78‐1.67)	1.27	(0.83‐1.96)
20 to <40	30	8005	64	1.03	(0.76‐1.39)	0.93	(0.65‐1.35)
≥40	41	3325	55	1.97	(1.40‐2.76)	1.56	(0.96‐2.54)
*p* for trend^d^				.004		.27	
Continuous, 10 y increments		38 814	360	1.22	(1.03‐1.45)	1.09	(0.92‐1.29)
**Time since cigarette smoking cessation (y)** ^g^							
Overall							
Never smokers		28 472	265	1	Reference	1	Reference
Stopped ≥20 y	25	7817	79	0.80	(0.59‐1.07)	0.91	(0.67‐1.23)
Stopped 10 to <20 y	14	8678	110	1.05	(0.81‐1.36)	1.06	(0.81‐1.38)
Stopped >0 to <10 y	5	8861	144	1.40	(1.10‐1.78)	1.26	(0.99‐1.62)
Current smokers	0	18 443	364	1.85	(1.52‐2.23)	1.67	(1.37‐2.03)
*p* for trend^d^				<.001		<.001	
Men							
Never smokers		5026	61	1	Reference	1	Reference
Stopped ≥20 y	25	5738	60	0.86	(0.58‐1.28)	0.90	(0.60‐1.34)
Stopped 10 to <20 y	14	6057	94	1.31	(0.91‐1.88)	1.21	(0.84‐1.74)
Stopped >0 to <10 y	5	5754	117	1.76	(1.24‐2.51)	1.44	(1.00‐2.07)
Current smokers	0	10 945	271	2.28	(1.66‐3.12)	1.88	(1.36‐2.59)
*p* for trend^d^				<.001		<.001	
Women							
Never smokers		23 446	204	1	Reference	1	Reference
Stopped ≥20 y	27	2079	19	1.10	(0.66‐1.83)	1.42	(0.82‐2.48)
Stopped 10 to <20 y	14	2621	16	0.77	(0.45‐1.33)	0.88	(0.51‐1.51)
Stopped >0 to <10 y	4	3108	27	1.08	(0.70‐1.66)	1.13	(0.73‐1.74)
Current smokers	0	7498	93	1.59	(1.21‐2.09)	1.59	(1.20‐2.11)
*p* for trend^d^				.005		.004	

Abbreviations: CI, confidence interval; CUP, cancer of unknown primary; HR, hazards ratio.

^a^ Analyses were adjusted for age at baseline (years) and sex.

^b^ Multivariable analyses were adjusted for age at baseline (years), sex and alcohol consumption (grams of ethanol per day).

^c^ Multivariable analyses of cigarette smoking status were additionally adjusted for frequency (N/d; continuous; centred) and duration of cigarette smoking (years; continuous; centred).

^d^ Tests for dose‐response trends were assessed by fitting ordinal variables as continuous terms in the Cox proportional hazards model.

^e^ Multivariable analyses of cigarette smoking frequency were additionally adjusted for current cigarette smoking and duration of cigarette smoking (years; continuous; centred).

^f^ Multivariable analyses of cigarette smoking duration were additionally adjusted for current cigarette smoking and frequency of cigarette smoking (N/d; continuous; centred).

^g^ Cigarette smoking cessation was additionally adjusted for the number of cigarette pack‐years (continuous; centred).

We observed that the more cigarettes a participant smoked, the higher the CUP risk (*P*_trend_ = .003). This trend was also observed in men (*P*_trend_ = .004). Overall, participants who smoked 10 to <20 or ≥20 cigarettes per day had a higher CUP risk (HR: 1.27, 95% CI: 1.00‐1.62 and HR: 1.42, 95% CI: 1.13‐1.80, respectively) compared to never smokers. There was no multiplicative interaction between cigarette smoking frequency, sex and CUP risk (*P*_interaction_ = .68).

Additionally, we noted that the longer a participant had smoked cigarettes, the higher the CUP risk (*P*_trend_ = .02). Participants who smoked cigarettes ≥40 years were at the highest risk of developing CUP (HR: 1.45, 95% CI: 1.09‐1.94) compared to never smokers. We found no multiplicative interaction between cigarette smoking duration, sex and CUP risk (*P*_interaction_ = .17).

Categories of cigarette smoking cessation were assessed in comparison to never smokers. Participants who stopped <10 years were at a higher CUP risk (HR: 1.26, 95% CI: 0.99‐1.62) compared to never smokers (*P*_trend_ < .001). A similar trend was observed in men (*P*_trend_ < .001) and in women (*P*_trend_ = .004).

We observed no multiplicative interaction between alcohol consumption, cigarette smoking frequency and CUP risk (*P*_interaction_ = .12) (see Table 5). However, we did find increased risks for most exposure combinations of alcohol consumption and cigarette smoking categories, for participants who smoked 10 to <20 or ≥20 cigarettes per day compared to abstainers and never smokers as the reference group. In addition, we found that participants who drank ≥30 g of ethanol per day and who smoked ≥20 cigarettes per day had the highest risk of developing CUP (HR: 2.87, 95% CI: 1.95‐4.22) compared to abstainers and never smokers. We also assessed whether there was interaction on additive scale between the highest exposure categories of alcohol consumption (≥30 g of ethanol per day), cigarette smoking (smoking ≥10 cigarettes per day) and CUP risk in comparison to the lowest exposure categories of alcohol consumption (<30 g of ethanol per day) and cigarette smoking (smoking <10 cigarettes per day). The RERI was 1.14 (95% CI: 0.33‐1.96); *P* = .006, which indicates that there is interaction on additive scale (see Table 6).

**TABLE 5:** Interaction of alcohol consumption, cigarette smoking and cancer of unknown primary risk (multivariable^a^‐adjusted incidence HRs) on multiplicative scale

	Alcohol consumption (g/d)				
	Abstainers	>0 to <5	5 to <15	15 to <30	≥30
Cigarette smoking frequency (N/d)		
Never smokers					
Cases	75	122	46	16	6
Person time at risk (y)	10 187	11 067	4802	1717	700
HR	1	1.52	1.20	1.17	0.96
95% CI	Reference	(1.11‐2.08)	(0.80‐1.80)	(0.65‐2.09)	(0.39‐2.38)
>0 to < 10					
Cases	13	32	40	21	13
Person time at risk (y)	2137	3742	3559	2219	864
HR	0.73	1.05	1.27	1.05	1.53
95% CI	(0.38‐1.41)	(0.66‐1.69)	(0.81‐1.99)	(0.61‐1.80)	(0.76‐3.08)
10 to <20					
Cases	49	47	67	58	43
Person time at risk (y)	2522	3653	4348	3151	1578
HR	1.87	1.25	1.61	1.65	2.15
95% CI	(1.19‐2.92)	(0.81‐1.93)	(1.09‐2.37)	(1.08‐2.52)	(1.32‐3.50)
≥20					
Cases	49	46	64	58	98
Person time at risk (y)	2335	3020	3505	4072	3165
HR	2.05	1.66	1.82	1.32	2.87
95% CI	(1.32‐3.18)	(1.09‐2.54)	(1.22‐2.73)	(0.87‐2.01)	(1.95–4.22)
*p* for interaction^b^					.115

Abbreviations: CI, confidence interval; HR, hazards ratio.

^a^ Adjusted for age at baseline (years), sex, current cigarette smoking and duration of cigarette smoking (years; continuous; centred).

^b^ *P* value for interaction between categories of alcohol consumption and cigarette smoking based on the Wald test and cross‐product terms in the Cox proportional hazards model.

**TABLE 6:** Interaction of alcohol consumption, cigarette smoking and cancer of unknown primary risk on additive scale

	Alcohol consumption	
	Low consumption (<30 g of ethanol)	High consumption (≥30 g of ethanol)
Cigarette smoking frequency		
Low exposure (smoking < 10 cigarettes per day)	1 (reference)	1.82 (1.59‐2.10)
High exposure (smoking ≥ 10 cigarettes per day)	1.31 (0.83‐2.08)	3.28 (2.70‐3.99)

*Note*: Measure of interaction on additive scale: RERI = 1.14 (95% CI: 0.33‐1.96); *P* = .006.

Abbreviations: CI, confidence interval; RERI, relative excess risk due to interaction.

Results from the sensitivity analysis with restriction to histologically verified CUP cases, and results after excluding the first 2 years and the first 5 years of follow‐up did not differ substantially from the findings of the complete multivariable analysis (data not shown).

## 4 DISCUSSION

In this prospective cohort study, alcohol consumption and cigarette smoking were found to be associated with CUP risk. Associations were increased for participants who drank ≥30 g of ethanol per day. Current smokers were at an increased risk of developing CUP. The more cigarettes (N/day) and the longer (years) participants had smoked, the greater their CUP risk. No multiplicative interaction was observed between alcohol consumption, cigarette smoking frequency and CUP risk. We did, however, find an interaction on additive scale between the highest exposure categories of alcohol consumption and cigarette smoking frequency and CUP risk.

Hitherto, only two prospective cohort studies have investigated the association between alcohol consumption and CUP risk. The European study, the European Prospective Investigation into Cancer and Nutrition (EPIC), includes 651 incident CUP cases. Results from the cohort revealed an increased CUP risk (HR: 1.42) for patients who consumed >60 g of ethanol per day compared to an intake of 0‐12 g/d, although not statistically significant.^10^ The Australian study is a prospective cohort study that compared 327 incident cancer registry‐notified CUP cases to two sets of controls that were randomly selected (3:1) using incidence density sampling with replacement. They observed no associations between alcohol consumption and CUP risk compared to the metastatic cancer controls and compared to the general cohort population controls.^11^ In the NLCS, we have found a positive association between alcohol consumption and CUP risk. The association was more pronounced in participants who drank ≥30 g of ethanol per day compared to abstainers. Additionally, our stratified analysis indicates that the CUP risk was especially increased in men. However, it should be noted that alcohol consumption categories differed between the European study, the Australian study and the NLCS, which makes it difficult to compare outcomes.

In an additional analysis in the European study, squamous cell carcinoma cases were deliberately excluded when assessing the association between alcohol consumption and CUP risk, because the majority of these cases had metastases in cervical lymph nodes, which could indicate the primary origin to be a tumour in the upper aerodigestive tract.^10^ After excluding squamous cell carcinoma cases from our cohort (N = 47), no notable changes were identified for the association between alcohol consumption and CUP risk (data not shown).

The European study demonstrated that current heavy smokers (26+ cigarettes per day) had an increased risk of developing CUP (HR: 3.66) compared to never smokers.^10^ Similarly, the Australian study reported that current smokers (odds ratio [OR]: 3.42) or ex‐smokers (OR: 1.95) were associated with CUP risk compared to never smokers.^11^ A Swedish case‐control study used data on 463 CUP patients, their study indicated that smoking was a risk factor for CUP (OR: 1.82) compared to no smoking.^16^ However, the exposure category of no smoking was not described in‐depth and possibly included ex‐smokers. In the NLCS, we also found current cigarette smokers to be at a greater risk of developing CUP (HR: 1.59) compared to never smokers. Although this association between smoking and CUP is weaker compared to those findings in the abovementioned studies, it should be noted that our study used different categories for measuring cigarette smoking. However, in accordance with the European study, we observed an association between smoking and CUP risk, which was elevated in the highest category of smoking frequency.^10^ In contrast, the Australian study observed no difference in risk associated with <20 or ≥20 cigarettes per day.^11^

The European study also reported that participants who had quit smoking ≤10 years ago were at a higher risk of CUP (HR: 1.34) than participants who had never smoked.^10^ In the NLCS, we found that participants who had stopped smoking <10 years were at a higher risk of developing CUP (HR: 1.26) compared to never smokers. Accordingly, our results are similar to those of the European study, which means our results are again in line with those of the European study.

Our study provides novel information on the association between cigarette smoking frequency, cigarette smoking duration and CUP risk. We found CUP risk to be more pronounced in the highest exposure categories of both cigarette smoking frequency and cigarette smoking duration, suggesting that the more the cigarettes (N/d) or the longer (years) the participants smoked, the greater their risk of developing CUP.

We found no multiplicative interaction effect between alcohol consumption, cigarette smoking frequency and CUP risk. However, we did find that participants who consumed the highest intake level of alcohol and smoked the highest number of cigarettes had a greater risk of CUP than either abstainers or never smokers. In addition, we found a significant additive interaction between the highest exposure categories of alcohol consumption and cigarette smoking frequency and CUP risk. This means that the combined effect of alcohol consumption and cigarette smoking frequency is larger than the sum of the individual effects of both alcohol consumption and cigarette smoking frequency.^25^ It should however be acknowledged that for assessing the interaction on additive scale, exposure categories were generated in a dichotomous manner.

### 4.1 Strengths and limitations

An important strength of our study is its prospective cohort design. A further strength is that the NLCS consists of a large cohort of 120 852 participants who were followed up for cancer incidence by the cancer registry in the Netherlands. Cases were registered by trained registry clerks who had access to the medical files and entered data by applying uniform coding rules. Furthermore, we were able to analyse 963 incident CUP cases, which is a much higher number of cases than previous studies have used to investigate CUP aetiology. It should, however, be acknowledged that the CUP definition used here might differ from that used in other countries because the criteria for defining “CUP” are heterogeneous. CUP cases within our study were consistently registered by NCR registry clerks, for which data were retrieved from pathology and clinical reports.^26^ Within the NLCS, information on alcohol consumption and exposure to smoking were collected before the outcome, minimising the effect of information bias. A potential limitation of the current study is that data on all exposure variables are self‐reported, which may have resulted in bias due to misclassification. However, we expect this misclassification to be nondifferential. Another potential limitation is that the questions regarding smoking behaviour are not validated. Even so, the questions included detailed categories that the participant could answer. Unfortunately, we do not have data to check which diagnostic methods were used for our CUP patients. Nevertheless, if we restrict our analysis purely to histologically verified CUP cases, for whom extended diagnostic methods are more likely, we find that the results do not differ greatly from the complete multivariable analyses. Accordingly, the findings from the complete multivariable analyses are representative of CUP cases with or without an extensive diagnostic work‐up.

## 5 CONCLUSIONS

In our study, alcohol consumption and cigarette smoking were found to be associated with an increased CUP risk. These findings suggest that lifestyle recommendations on cancer prevention regarding not drinking alcohol and avoiding exposure to smoking are also valid for CUP.

## CONFLICT OF INTEREST

There are no competing financial interests in relation to this work.

## ETHICS STATEMENT

The institutional review boards of the Netherlands Organisation for Applied Scientific Research TNO (Zeist) and Maastricht University (Maastricht) approved the execution of the NLCS. Participants agreed to be included into the cohort and follow‐up by returning the questionnaire they completed.

## Data Availability

The datasets generated and/or analysed during the current study are not publicly available because the informed consent does not allow for that. However, anonymous data that are minimally required to replicate the outcomes of the study will be made available upon reasonable request and approval by the institutional review boards.
